# Cardiac impalement injury by a steel rebar: A case report

**DOI:** 10.1016/j.ijscr.2019.12.006

**Published:** 2019-12-16

**Authors:** Byungsu Yoo, Yoon Cheol Shin

**Affiliations:** Department of Thoracic and Cardiovascular Surgery, Inje University Ilsan Paik Hospital, Republic of Korea

**Keywords:** Cardiac injury, Impalement, Steel rebar, Computed tomography

## Abstract

**Introduction:**

Penetrating cardiac injury often occurs in South Korea. However, impalement injury of the heart with other organs is rare and fatal. We present a case of cardiac impalement injury by a steel rebar.

**Presentation of case:**

A 38-year-old man was brought to the emergency room with a steel rebar of 45.5 cm in length in situ. Chest tomography revealed that the rebar had entered the thorax to the right diaphragm via the left shoulder while piercing the heart. The patient was immediately taken to the operating room for surgery without removing the bar. We were able to rescue the patient with an emergency operation wherein the bleeding site was treated by pulling the steel rebar out step-by-step while performing cardiopulmonary bypass.

**Discussion:**

Early diagnosis and rapid surgery are important factors for patients with cardiac impalement injury. Haemostasis must be achieved patiently step- by- step without removing the foreign object at once. CPB is an appropriate treatment, even if there is a risk of bleeding.

**Conclusion:**

This case highlights the importance of immediate surgical treatment for the survival of severe thoracic impalemant injury patients.

## Highlights

• Cardiac impalement injuries may be fatal.
• We report a patient with cardiac injury due to penetration by a steel rebar.
• The patient also sustained pulmonary injuries.
• The steel rebar was pulled out gradually during cardiopulmonary bypass (CPB).
• CPB is an effective surgical treatment even if there is a risk of bleeding.

## 1 Introduction

Impalement injuries due to large objects or foreign objects penetrating the body cavity or limbs are severe and can be fatal. These injuries are often caused during traffic accidents, aircraft crashes, and accidents at construction sites, resulting in blunt and penetrating trauma.

Impalement injuries to the chest cause immediate death [1]. Survivors must be quickly transported to a hospital where proper surgery and treatment may save their lives. In such cases, the medical personnel are generally not aware of the extent of the injury and do not have sufficient time to evaluate the condition and perform resuscitation. Therefore, management of such cases presents a challenge to the medical staff.

Here, we report a case involving a patient who sustained injuries when a with steel rebar penetrated through the left shoulder and entered the right pleural cavity by piercing the left lung, left pulmonary artery, and right atrium and was successfully managed by emergency surgery with cardiopulmonary bypass.

This paper is written according to the SCARE criteria [1].

## 2 Presentation of case

**Fig. 1:**
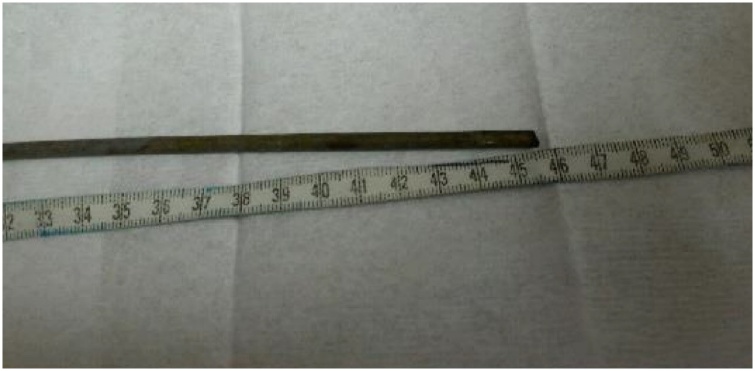
The steel rebar of 45.5 cm in length and 6 mm in diameter removed by surgery.

A 38-year-old man was transferred to the emergency room due to a steel rebar penetrating through the left shoulder. The rebar was 6 mm in diameter and 45.5 cm in length (Fig. 1). As he was too intoxicated to talk, we were informed of the injury by his friend, did not witness the accident but found the patient with the steel rebar penetrating his body. The patient was first taken to the local hospital and then transported to our hospital for surgery.

**Fig. 2:**
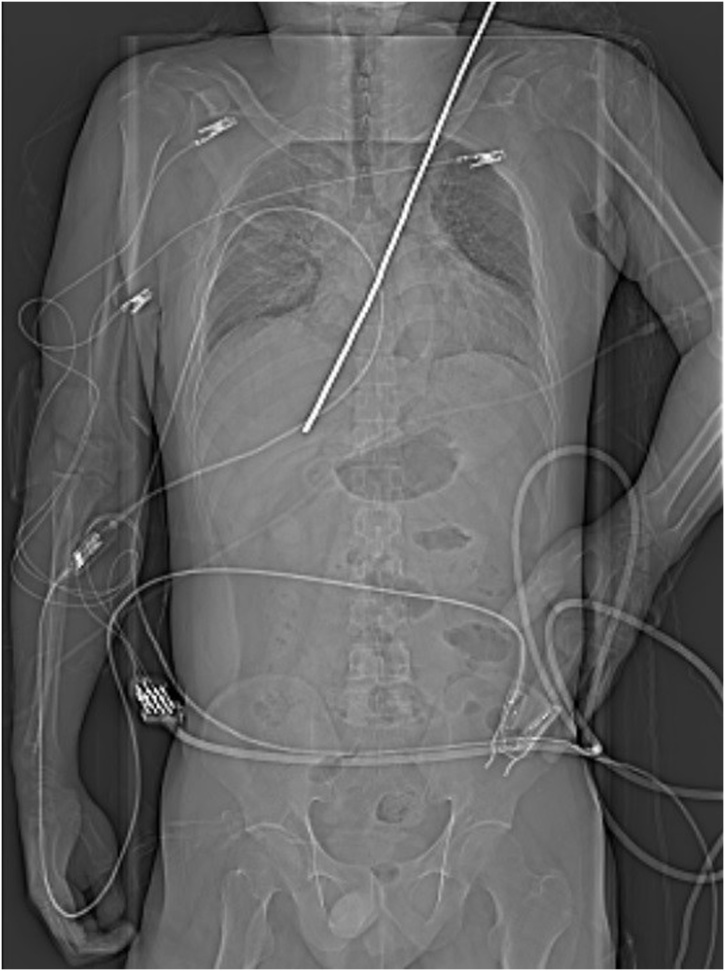
Chest computed tomography scan showing the steel rebar penetrating through the left shoulder into the right atrium.

**Fig. 3:**
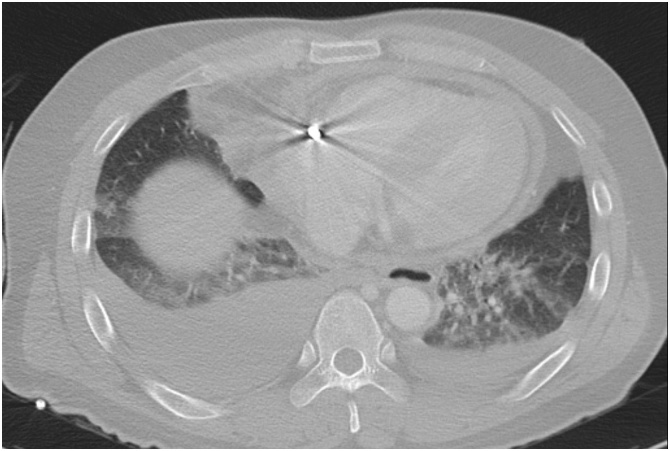
Chest computed tomography scan CT showing hemopericardium and bilateral haemothorax.

The vital signs were unstable, and chest computed tomography (CT) revealed that the steel rebar had penetrate through the left shoulder, left lung, left pulmonary artery, and right atrium (Fig. 2). Hemopericardium and bilateral haemothorax were also observed (Fig. 3). We did not hesitate to perform emergency surgery on the patient.

With general anaesthesia, a sternotomy was performed with the rebar steel in situ. There was a small amount of blood in the pericardium. After bicaval cannulation, cardiopulmonary bypass (CPB) was started. Both the pleural cavities were opened, and the hematoma was removed. We did not arrest the heart. The volume amount of haemothorax on both sides was approximately 1500 ml. We then evaluated how the steel rebar damaged the various organs. The steel rebar punctured the left lung, left pulmonary artery, and right atrium. Fortunately, there was no liver damage because the rebar steel did not pierce the diaphragm. After the snaring of both caval veins, we opened the right atrium and slowly pulled out the steel rebar. Two right atrial tears were identified and repaired after irrigation. After complete haemostasis of the right atrium was achieved, the steel rebar was pulled gradually. Bleeding from the left pulmonary artery was then noticed. We irrigated the injury site and repaired it.

Finally, the steel rebar was removed, and the injured lung was repaired. There was minimal bleeding from the injury site on the left shoulder. The shoulder wound was irrigated and closed. CPB support was weaned and removed. The total CPB time was 107 min. The pericardium and bilateral pleural cavities were irrigated. After the chest tubes were inserted, the pericardium was closed partially. We wired the sternum and closed the wound layer by layer. The total operation time was 4 h. The patient’s vital signs were stable after surgery, and he was transferred to the cardiac intensive care unit (CICU).

The patient was stable and extubated 12 h after the operation. He was transferred to the general ward and discharged 12 days after the surgery. He has had no complications and no sequelae associated with the surgery and was healthy till the last follow-up.

## 3 Discussion

We report a patient with injuries to the heart and other internal organs due to impalement by a steel rebar who was successfully treated by CPB. Cardiac trauma may be classified as non-penetrating (referred to as blunt cardiac injury) and penetrating injuries. Cardiac penetrating injuries are rare but are more dangerous and fatal. The reported pre-hospital mortality rate of cardiac penetrating injury was 94%, and the in-hospital mortality rate was 50% [1]. Early diagnosis and immediate surgery are essential for patient survival. The mortality rate increased on combined non-cardiac damage [2,3].

It is imperative that surgery is not delayed and is performed before the blood pressure is elevated. Treatment must be administered in the hope of reviving even a dying patient even when there is cardiac arrest or the absence of detectable blood pressure. A high survival rate can be obtained when emergency surgery is performed without hesitation [3]. In our case, the time to surgery was 1 h.

In most patients with penetrating cardiac injuries arriving at the hospital, bleeding may have ceased or may have been relieved by the compression of the cardiac wound due to hypotension, coagulation, cardiac tamponade, and haemopneumothorax. To ensure that this balance is not disrupted before surgery, Gao et al. proposed three principles [4]. First, excessive transfusion should be avoided [5]. Second, preoperative pericardiocentesis should not be performed. Haemorrhage might recur due to decompression and clot dislodgement and may cause iatrogenic injuries [6]. Third, to prevent tension pneumothorax under positive pressure anaesthesia, intercostal tube drainage must be performed. Moderate pericardial tamponade can temporarily stop bleeding from the cardiac injury sites with relatively low risk of cardiac arrest [5]. Cardiac tamponade is the most effective independent predictor of survival that reduces mortality [3,7]. In our case, there was no cardiac tamponade.

Some survival benefits of CPB have been noted in selected cases. CPB is useful for cardiac injury surgery, although there is a risk of the increased bleeding tendency due to systemic heparinization [8]. CPB also restores and correct metabolic defects [9]. CPB indications are coronary artery injuries, valvular injuries, large intracardiac septal defects, retained intracardiac projectiles, and coronary-cameral fistula [10].

In cases of cardiac impalement injury, the foreign objects should be maintained in its place till the bleeding site is identified and the haemostasis is achieved. The removal of foreign objects may eliminate the tamponading effect on the main vessel or body cavities, which may result in massive or fatal bleeding. The foreign objects can also damage the surrounding tissues and aggravate injuries [4,11].

We think that by pulling the steel rebar out step-by-step with CPB, we were able to perform a successful surgical intervention for the treatment of the injuries.

## 4 Conclusions

Our experience with this case suggests that, early diagnosis and rapid surgery are important factors affecting the survival of patients with cardiac impalement injury. Haemostasis must be achieved patiently step- by- step without removing the foreign object at once. CPB is an appropriate treatment, even if there is a risk of bleeding.

## Sources of funding

There is no funding to declare.

## Ethical approval

This paper was approved by the Institutional Review Board of Ilsan Paik Hospital.

IRB File No.: 2019-04-016.

## Consent

The director of my department confirms that he has taken responsibility that exhaustive attempts have been made to contact the family and that the paper has been sufficiently anonymised not to cause harm to the patient or their family. A copy of this signed document is available for review by the Editor-in-Chief of this journal on request.

## Author contribution

Yoon Cheol Shin, MD: study concept, data collection.

Byungsu Yoo, MD: writing the paper.

## Registration of research studies

This is not a human study but a case report.

## Guarantor

Yoon Cheol Shin, MD.

Byungsu Yoo, MD.

## Provenance and peer review

Not commissioned, externally peer-reviewed.

## Declaration of Competing Interest

There are no conflicts of interest to declare.
